# Isovalerylspiramycin I suppresses non-small cell lung carcinoma growth through ROS-mediated inhibition of PI3K/AKT signaling pathway

**DOI:** 10.7150/ijbs.69989

**Published:** 2022-05-21

**Authors:** Zeyu Liu, Moli Huang, Yue Hong, Shaoyang Wang, Yongle Xu, Cheng Zhong, Jingyuan Zhang, Zhengping Zhuang, Shan Shan, Tao Ren

**Affiliations:** 1 Department of Respiratory and Clinical Care Medicine, Shanghai Jiao Tong University Affiliated Sixth People's Hospital, Shanghai, 200233, China.; 2 Department of Bioinformatics, School of Biological and Basic Medical Sciences, Soochow University, Suzhou, 215123, China.; 3 Stem Cell Center, Shanghai Jiao Tong University Affiliated Sixth People's Hospital, Shanghai, 200233, China.; 4 Neuro-Oncology Branch, Center for Cancer Research, National Cancer Institute, National Institutes of Health, Bethesda, MD 20892, USA.; 5 Surgical Neurology Branch, National Institute of Neurological Disorders and Stroke, National Institutes of Health, Bethesda, MD 20892, USA.; 6 Shanghai Key Laboratory of Sleep Disordered Breathing, Shanghai Jiao Tong University Affiliated Sixth People's Hospital, Shanghai, 200233, China.

**Keywords:** Isovalerylspiramycin I (ISP-I), Non-small cell lung cancer (NSCLC), apoptosis, G2/M arrest, ROS, PI3K/AKT signaling pathway

## Abstract

Novel drugs are required for non-small cell lung cancer (NSCLC) treatment urgently. Repurposing old drugs as new treatments is a practicable approach with time and cost savings. Some studies have shown that carrimycin, a Chinese Food and Drug Administration (CFDA)-approved macrolide antibiotic, possesses potent anti-tumor effects against oral squamous cell carcinoma. However, its detailed component and underlying mechanisms in anti-NSCLC remain unknown. In our study, isovalerylspiramycin I (ISP-I) was isolated from carrimycin and demonstrated a remarkable anti-NSCLC efficacy in vitro and in vivo with a favorable safety profile. It has been proven that in NSCLC cell lines H460 and A549, ISP-I could induce G2/M arrest and apoptosis, which was mainly attributed to ROS accumulation and subsequently PI3K/AKT signaling pathway inhibition. Numerous downstream genes including mTOR and FOXOs were also changed correspondingly. An observation of NAC-induced reverse effect on ISP-I-leading cell death and PI3K/AKT pathway inhibition, emphasized the necessity of ROS signaling in this event. Moreover, we identified ROS accumulation and PI3K/AKT pathway inhibition in tumor xenograft models in vivo as well. Taken together, our study firstly reveals that ISP-I is a novel ROS inducer and may act as a promising candidate with multi-target and low biological toxicity for anti-NSCLC treatment.

## Introduction

Lung cancer is the second most commonly diagnosed cancer worldwide, causing over 1.8 million deaths in 2020 1. Although diagnostic and therapeutic methods have markedly improved, the prognosis of lung cancer continues to be poor with a 5-year survival rate of only 23% 2. NSCLC is the most prevalent histological subtype of lung cancer, accounting for 85-90% of all cases 3. The first choice of treatment for NSCLC is surgical removal, however, high risk of postoperative recurrence is still the biggest challenge and associates with significantly reduced survival 4. Besides, a considerable number of patients with advanced NSCLC are inoperable 5. Chemotherapy and radiotherapy are still the basic anticancer strategies in the treatment of lung cancer 6, yet its efficacy and adverse side effects issues occur frequently and result in cancer recurrence and lethal outcomes 7. In recent years, the emerging immunotherapy and molecular targeted therapy have made great contributions to the precision NSCLC treatment. Nevertheless, only a small fraction of NSCLC patients can benefit from these two approaches due to its exactly genetic mutation matching and high prices 8-11. In addition, emergence of drug resistance has limited the benefit of immunotherapy and molecular targeted therapy, especially single-target inhibitors; thus, they cannot provide long-term survival for most patients 10, 11. Consequently, novel anticancer agents to inhibit NSCLC with low biological toxicity and multi-target are urgently needed.

Recent studies have shown that tumor cells possess a higher level of ROS and are easier to reach the toxic threshold, thus, further ROS accumulation can act as an effective means of tumor inhibition while normal cells are less or not affected 12. The overproduction of ROS in NSCLC is served as an initiator and can trigger multiple pathways alterations related to apoptosis, cell cycle and protein synthesis. One prototypical example is the PI3K/AKT signaling pathway 13. Numerous genes (PIK3CB, AKT1, MTOR, FOXOs, CDKN1A etc.) is involved in PI3K/AKT signaling pathway and can induce expression and activation of multiple receptor tyrosine kinases (RTKs) 14. Each step in the RTK cascade can be a potential therapeutic target and multi-targeted RTK inhibitors are clinically effective against tumors 15. Taken together, novel ROS inducer may act as a promising attempt in the development of anti-tumor agent with multi-target and low biological toxicity.

Developing new drugs is a very expensive and time-consuming process 16. In recent years, “new uses of old drugs” gets great attentions and provides an abundant source for novel anti-tumor agents 17. Carrimycin is a CFDA-approved newly synthesized macrolide antibiotic, which possesses excellent bioavailability and biosafety in phase I to III clinical trials. Previous studies have revealed that carrimycin can regulate cell physiology, proliferation and immunity through inhibition of protein synthesis, and exhibits a potential anti-tumor activity against oral squamous cell carcinoma 18. Accordingly, we hypothesize that carrimycin may have the properties of anti-NSCLC, which needs further explore.

To exactly clarify the pharmacological mechanism of carrimycin, we isolated and purified the active component of carrimycin, Isovalerylspiramycin I (ISP-I). The efficacy of ISP-I on the inhibition of NSCLC and its underlying mechanisms were evaluated in the current research. We revealed that ISP-I significantly inhibited NSCLC growth both in vitro and in vivo via excessive ROS accumulation, which mediated PI3K/AKT signaling pathway inhibition. In summary, our study reveals that ISP-I is a novel ROS inducer and may be a promising candidate with low biological toxicity and multi-target ability for anti-NSCLC treatment.

## Materials and methods

### Reagents and antibodies

ISP-I (95.9% purity) was procured from Shanghai Tonglian Pharmaceutical Co., Ltd. (China). For cell experiments, ISP-I was dissolved to 100mM stock concentration in DMSO (Sigma) and diluted to appropriate concentrations with cell culture medium (final DMSO concentration in culture medium was no greater than 0.015%). For animal experiments, ISP-I was dissolved in Polyethylene glycol (PEG; Sigma), vegetable oil and Tween-80 (Sigma) mixed solvents (v:v:v = 9.5:9.5:1) at 24mg/ml, and then diluted to the final concentration with sterilized water.

The CCK-8 solution, DCFH-DA and crystal violet solution were all procured from Beyotime (Shanghai, China). Dihydroethidium (DHE, #D7008) and N-acetyl-l-cysteine (NAC, A9165) were provided by Sigma (MO, USA). The antibodies used in our research were provided in Supplementary Table S1.

### Cell lines and culture

Human NSCLC cell lines (H460 and A549) as well as human lung epithelial cells (Beas2B) were acquired from Chinese Academy of Sciences cell bank. Beas2B was grown in DMEM (Gibco, Grand Island, NY, USA) while H460 and A549 were grown in RPMI-1640 (HyClone, Logan, UT, USA) at 37 °C in a humidified 5% CO_2_ environment. Media were both supplemented with 10% fetal bovine serum (FBS, HyClone, Logan, UT, USA) and streptomycin/penicillin (1%, Gibco, Grand Island, NY, USA). A Nikon Eclipse Ts2 inverted microscope (100× magnification) was used to observe and image cell morphology and numbers.

### CCK-8 assay

Cells were seeded in 96-well plates at 4 × 10^3^ cells/well followed by overnight incubation. Next, they were treated with different doses of ISP-I or DMSO (control) for 24, 48 and 72 h. NSCLC cells after the different treatments were incubated with CCK-8 solution (1:10 dilution) for 1h at 37°C. Fluorescence for every plate was measured by SpectraMax® absorbance reader (Molecular Devices) at 450 nm. The half-maximal inhibitory concentrations (IC50) were evaluated by GraphPad Prism version 8.3.0 software.

### Colony formation assay

Cells cultured in 6-well plates (10^3^ cells/well) were allowed to attach followed by overnight incubation and treatment with varying of ISP-I or DMSO (control) doses. After different treatments for 24 h, drug-comprising media were exchanged with complete culture media. Medium replacement was done after every 3 days until the surviving cells formed visible colonies 14 days later. Then, we stained the colonies with crystal violet (0.5%, 20 min) and washed thoroughly. Visible colonies with ≥ 50 cells were enumerated.

### Cell apoptosis and cell cycle assay

Cells in 6-well plates (2 × 10^5^ cells/well) were incubated overnight, then treated with 5μM, 10μM, 15μM of ISP-I or DMSO (control) for 48h. Apoptosis was estimated by the Annexin V-FITC/PI Apoptosis Kit (AP101, MULTI Sciences), and was detected by flow cytometry (CytoFLEX LX; Beckman Coulter, Inc., USA). Operations were carried out according to kit instructions. Data were processed by CytExpert software (Beckman Coulter, USA). Cell cycles were evaluated by the Cell Cycle Assay Kit (CCS012, MULTI Sciences) and measured using flow cytometry (BD Accuri C6 flow cytometer, BD Biosciences, USA). Results were analyzed by ModFit software.

### RNA-sequencing analysis

Total RNA extraction from non-treated as well as ISP-I (10μM) treated NSCLC cells (3 replicates per group) was done by TRIzol reagent (Invitrogen, Carlsbad, CA, USA) and RNeasy Mini Kit (Qiagen, Valencia, CA, USA). The concentrations, quality as well as integrity of extracted RNA were assessed by NanoDrop spectrophotometry (Thermo Scientific). Then, 3 micrograms of RNA (RIN 10.0) from each sample were utilized as input materials for preparation of RNA samples. Samples were sequenced and analyzed by Shanghai Personal Biotechnology Cp. Ltd. Briefly, sequencing libraries were constructed by TruSeq RNA Sample Preparation Kit (Illumina, San Diego, CA, USA), quantified using qRTPCR and the Agilent high sensitivity DNA assay on Bioanalyzer 2100 system (Agilent), and then sequenced on Illumina Novaseq 6000 system to generate 150bp pair-end reads. The reads, filtered by FastQC, were mapped to human genome (hg38) using HISAT2 software. Read counts were calculated using HTSeq v0.9.1 and output as FPKM upon normalization. Differentially expressed genes (DEGs) were defined as fold change > 2 and normalized p < 0.05. Heatmap of DEGs was drawn by R. Gene Ontology (GO) (http://www.geneontology.org/) was used for analysis biological processes (BP) while the Kyoto Encyclopedia of Genes and Genomes (KEGG) for pathway analysis. DEGs were subjected to GO_BP enrichment, KEGG analysis as well as Gene Set Enrichment Analysis (GSEA). Circle plots were drawn to depict the related DEGs involved in biological process terms of interest. Raw data and processed data were uploaded to GEO database (accession number: GSE 200370).

### Quantitative real-time PCR analysis (qRTPCR)

Total RNA was prepared as mentioned above, after which 1μg of isolated RNA was utilized in reverse transcription assays using a RevertAid First Strand cDNA Synthesis Kit (Thermo Scientific) as indicated by the manufacturer.

RT-PCR analysis were performed in a reaction volume of 20μl, including 10ng of cDNA, 200 nM each of forward as well as reverse primer, 1x Power SYBR Green Master Mix (Applied Biosystems) and ddH2O, followed by amplification using the QuantStudio 5 System (Applied Biosystems). Supplementary Table S2 showed the primers used in this study. Final normalized expression values were analyzed using 2-△△CT method relative to the endogenous control GAPDH.

### Measurement of ROS

Cells were seeded at 2 × 10^5^ cells/well in 6-well plate and 10^5^ cells/dish in a 35-mm glass bottom dish (Nest, Shanghai). After an overnight culture, 10μM ISP-I or DMSO (control) with or without 2mM NAC (a ROS scavenger) was supplemented to cells and incubated for 48 h 19-21. Treated cells were then loaded with DCFH-DA (1:1000 dilution) and incubated at 37 °C for 30 min. Removal of excess DCFH-DA was performed by washing with RPMI 1640 medium. Cell esterases could hydrolyze DCFH-DA to form DCFH, which was oxidized to fluorescent DCF by intracellular ROS 22. Fluorescent intensity of DCF was evaluated by flow cytometry, which was performed on Beckman CytoFLEX LX, followed by analysis using CytExpert software. DCF fluorescent intensity was also visualized by confocal microscopy (Olympus IX83, Tokyo, Japan) with an excitation laser at 473 nm and power at ~0.1 mW level. Image J was used to measure mean fluorescence intensity (MFI).

### Western blotting

Total protein extraction from cells was done by the RIPA buffer (#9806, Cell Signaling technology) supplemented with 1× Halt™ protease and protein phosphatase inhibitor cocktail (#78440, Thermo Scientific), and tumor tissue protein was obtained using tissue protein extraction kit (PC201, Epizyme) according to the instruction manuals. The Separation of equivalent amounts of proteins (20µg) was done using different concentrations (6%, 10%, 12.5%) of SDS-PAGE gel (PG110, PG112, Epizyme), and transferred to PVDF membrane, followed by blocking with QuickBlock™ Blocking Buffer (P0252, Beyotime). Then, they were washed thoroughly with TBST and incubated at 4 °C immersed with the primary antibodies overnight. Membranes were washed thrice and then incubated with secondary antibodies (2 h, 25°C). NcmECL Ultra (P10100A, P10100B, New Cell &amp) were used to visualize immunoreactive proteins via Bio-Rad ChemiDoc Imaging system.

### Xenograft tumor model

5×10^6^ H460 and A549 cells were subcutaneously administered into right flanks of male nude mice (5 weeks old, Shanghai South Model Biotechnology Co., Ltd.). When tumors reached approximately 100mm^3^, tumor-bearing mice were grouped randomly (n = 5/group) for daily oral gavage of ISP-I (30 or 60 mg/kg, experimental groups) or vehicle (water containing PEG, vegetable oil and Tween-80, control group) for 18days. Tumor sizes were determined every three days, and were calculated with the formula V = π/6(length×width^2^). When the largest tumors approached 2000 mm^3^ (day18, endpoint), mice were sacrificed, and tumors were harvested and weighed.

### Hematoxylin and eosin (HE) and immunohistochemistry (IHC) staining

Tumor tissues and organs of each group were fixed, paraffin-embedded, and sectioned into three-micrometer-thick. Sections were then deparaffinized in xylene after which decreasing ethanol concentrations were used for rehydration. HE staining was conducted following standard protocols for routine analysis. Briefly, staining of deparaffinized sections with hematoxylin was done for 5 min followed by dipping in 1% of acid ethanol and 1% ammonia water. Then, they were eosin-stained for 5 min. For IHC staining, after antigen repair with tetra-acetic acid (EDTA) buffer (PH8.0), sections were incubated in H2O2 solution (3%) to block endogenous peroxidase. Next, they were incubated with primary antibody (Ki-67, 1:200; cleaved caspase-3, 1:100) overnight at 4 °C and then with secondary antibody (HRP-conjugated goat anti-rabbit antibody, 1:100) for 2 h at RT. Immunohistochemical reactions were developed in a substrate solution of DAB and nuclei were counter-stained by hematoxylin. At last, slides were dehydrated with graded alcohol and cleared in xylene. The brown granules in the nucleus or cytoplasm represented positive expression of Ki-67 as well as cleaved caspase-3. The slides were observed and photographed under light microscopy (Olympus IX53, Tokyo, Japan).

### Dihydroethidium (DHE) staining

A portion of the tumor tissue from different groups were immediately frozen in OCT embedding agent (Sakura, 4583), and then cut into 6μm thick frozen sections by Thermo FINESSE 325. The frozen tumor tissue sections were next incubated with 20μM DHE (37°C, 30 min) in darkness. Afterwards, sections were thoroughly washed and photographed by a fluorescence microscope (Olympus BX53, Tokyo, Japan).

### Blood biochemical analysis

ISP-I (30 or 60 mg/kg, experimental groups) or vehicle (water containing PEG, vegetable oil and Tween-80, control group) were administered daily by oral gavage in nude mice. After 18 days, blood samples were collected from the mice of above groupings by removing eyeballs for serum biochemical analysis. The fresh blood samples were clotted for 2 h at room temperature, and then centrifuged (1000× g, 15 min) at 4°C to obtain serum. 200 μL serum was aliquot and analyzed of liver and kidney function indicators including alanine transaminase (ALT), aspartate transaminase (AST), albumin (ALB), blood urea nitrogen (BUN) and creatinine (Cr) by an automated Chemray 240 clinical analyzer (Rayto, Shenzhen, China).

### Orthotopic lung tumor model

To directly detect the efficacy of ISP-I against NSCLC, we generated orthotopic lung tumors in nude mice by tail vein injection with 1 × 10^6^ H460 or A549 cells. After tumor formation in the lung was ensured using a Skyscan-1176 microCT scanning, we divided the tumor-bearing mice into three groups (4 per group) randomly for daily oral gavage of ISP-I (30 or 60 mg/kg) or vehicle as mentioned above. Mice were sacrificed after 18 days of treatment and lungs from each mouse were taken out for HE staining. The number and size of tumors in the lung were evaluated by gross and histology analysis.

All animal procedures in this study were conducted under IACUC guidelines and approved by the Ethics Committee of Shanghai Jiao Tong University affiliated Sixth People's Hospital.

### Statistical analysis

All results were expressed as mean ± standard deviation (S.D.). Mean values were calculated from data obtained from three independent experiments, each performed in triplicate unless otherwise mentioned. Unpaired Student t test (group=2) and one way ANOVA followed by Dunnett's multiple comparisons test (group≥3) were used to compare the differences between groups. All statistical analysis were done by GraphPad Prism software 8.3.0. P < 0.05 was the cut-off for statistically significant.

## Results

### ISP-I suppressed the proliferation of NSCLC cells

**Fig 1:**
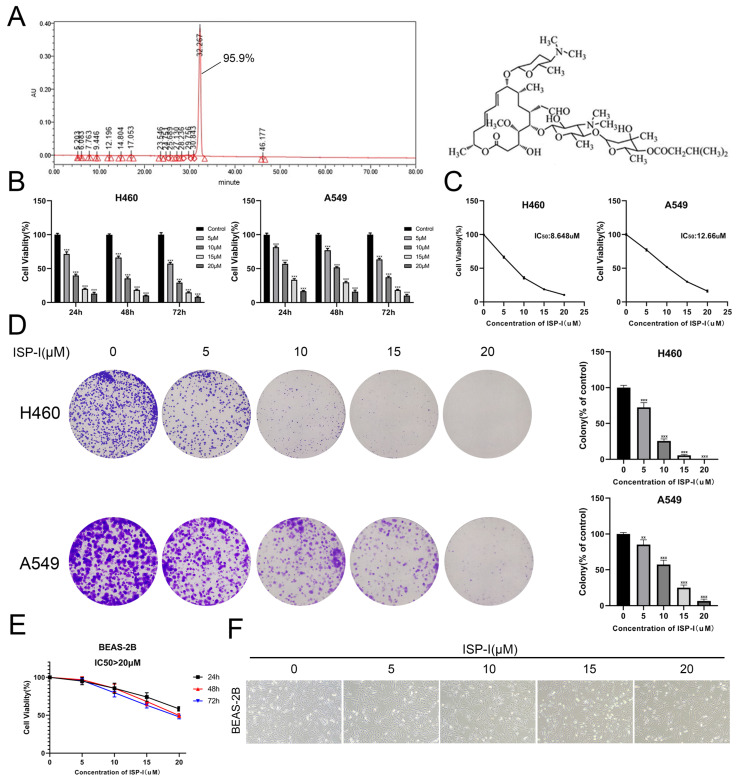
** SP-I suppressed NSCLC cell proliferation. (A)** The purity and chemical structure of ISP-I. **(B)** Cell viabilities of H460 and A549 treated with various doses of ISP-I were assessed by CCK-8 in 96-well plates. **(C)** IC50 values of ISP-I in H460 and A549 cells were determined after 48 h incubation by GraphPad Prism software. **(D)** Morphology and clonogenicity of H460 and A549 cells were visualized after crystal violet staining. Colonies were counted.** (E)** BEAS-2B were treated with varying doses of ISP-I at different durations. CCK-8 assay was performed for cell viability.** (F)** Morphology and number of BEAS-2B cells were observed under inverted microscope after 48h treatment with various dosages ISP-I. ** p < 0.01 and *** p < 0.001.

Purity and chemical structure of ISP-I were shown in Fig. 1A. Fig. 1B, C, shows that ISP-I dose- and time-dependently reduced cell viabilities. The IC50 doses of ISP-I for H460 and A549 were 8.648μM and 12.66μM, respectively (Fig. 1C). Then, we treated NSCLC cell lines with ISP-I for 24 h and evaluated colony formation abilities and growth 2 weeks later. It was revealed that colony formation was dose-dependently inhibited by ISP-I (Fig. 1D). Moreover, to evaluate cell toxicity effects of ISP-I in human normal lung epithelial cells, we next treated BEAS-2B with ISP-I. The IC50 doses of ISP-I for BEAS-2B were above 20 μM (Fig. 1E), which was greater than its efficacy doses in NSCLC cells. The cell morphology and number of NSCLC cells (Supplementary Fig. S1) and BEAS-2B cells (Fig. 1F) were observed after treatment with ISP-I at various dosages for 48h. These findings imply that ISP-I toxicity was selective to NSCLC cells, when compared to normal cells.

### ISP-I induced apoptosis and G2/M arrest in NSCLC cells

**Fig 2:**
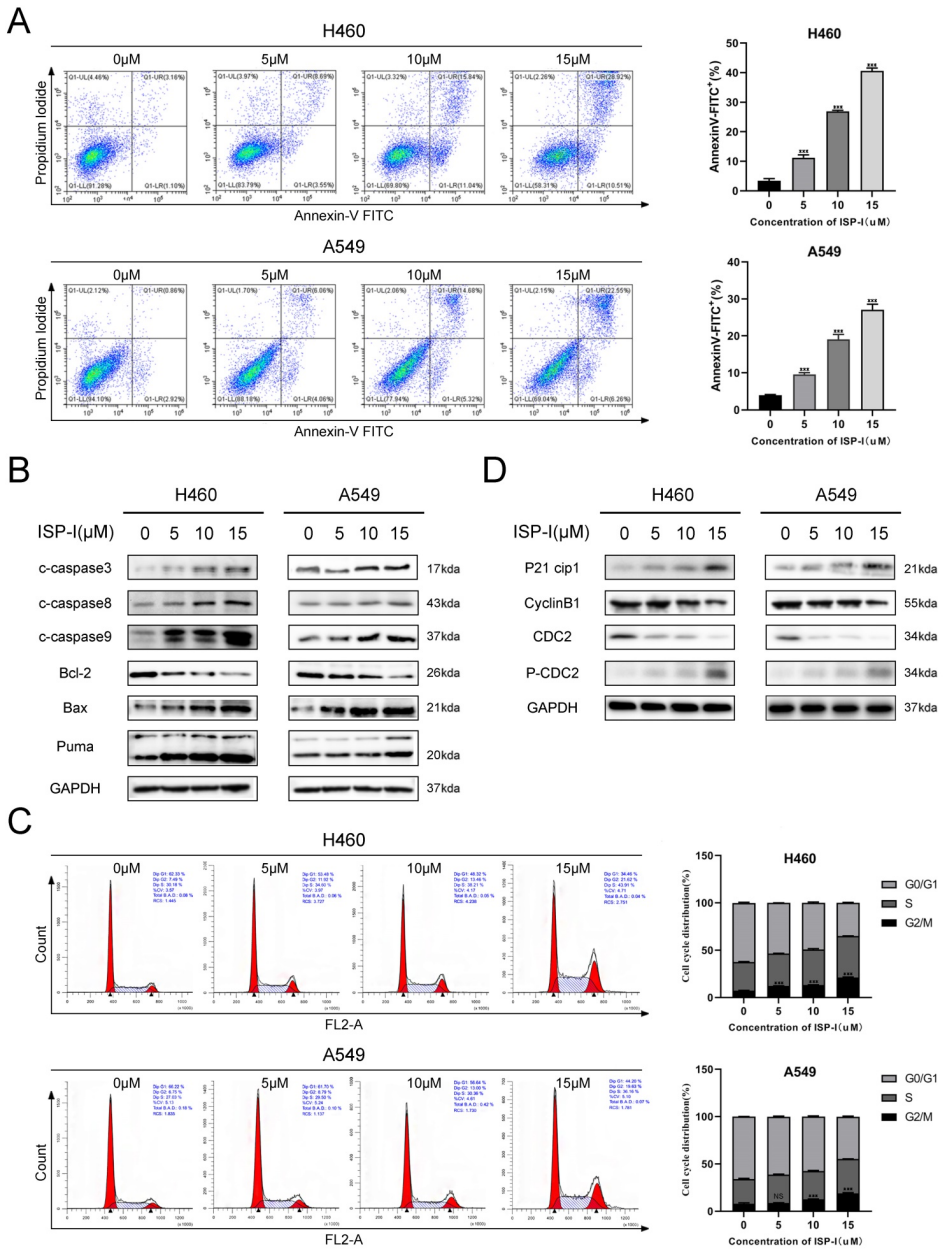
** ISP-I induced apoptosis and G2/M arrest in H460 and A549. (A)** NSCLC cells were exposed to increasing doses of ISP-I for 48h, and apoptosis was evaluated by flow cytometry analysis after Annexin V and PI double staining.** (B)** Bax, Bcl-2, Puma and cleaved Caspase 3/ 8/ 9 expression levels were evaluated when H460 and A549 were treated with ISP-I at 0, 5, 10 and 15 μM for 48h. **(C)** Cell cycle analysis after treatment for 48 h with increasing dosages of ISP-I. **(D)** Protein levels of p21, phospho-cdc2, cdc2 and cyclin B1 were investigated after treatment with increasing doses of ISP-I for 48 h by western blotting. ns p > 0.05, * p < 0.05, ** p < 0.01 and *** p < 0.001.

We analyzed apoptosis and cell cycle in H460 and A549 to further determine the details of ISP-I induced cytotoxicity in NSCLC. Here, we revealed that 48 h treatment with ISP-I at a gradually increased dose of 5μM, 10μM and 15μM induced 11.22%, 26.95%, 40.55% apoptosis in H460 and induced 9.54%, 19.01%, 27.09% apoptosis in A549 cells (Fig. 2A). To additional validate our cell apoptosis data, we checked mitochondria-induced endogenous apoptosis associated genes BAX, BBC3 (Puma) and BCL2 expression levels 23, and also examined activation of caspase-9 (endogenous apoptosis initiator), caspase-8 (exogenous apoptosis initiator) and caspase-3 (apoptosis executioner) in the treated samples (Fig. 2B). These results implied that ISP-I induced both endogenous apoptosis and exogenous apoptosis meanwhile.

With regards to the mechanism of anti-proliferative effects of ISP-I, Fig. 2C showed that ISP-I treatment for 48h obviously ascended the abundance of cells in G2/M phase. The transition from G2 phase to mitosis was initiated by activated cyclin B1/cdc2 complex, which could be inhibited by the increased p21 24, 25. As shown in Fig. 2D, ISP-I elevated phospho-cdc2 and p21 whilst suppressing cyclin B1 and cdc2 protein levels. Collectively, the above results indicated that ISP-I caused G2/M arrest by altering vital molecules during G2/M phase transitions.

### The anti-tumor effect of ISP-1 depended on ROS accumulation

**Fig 3:**
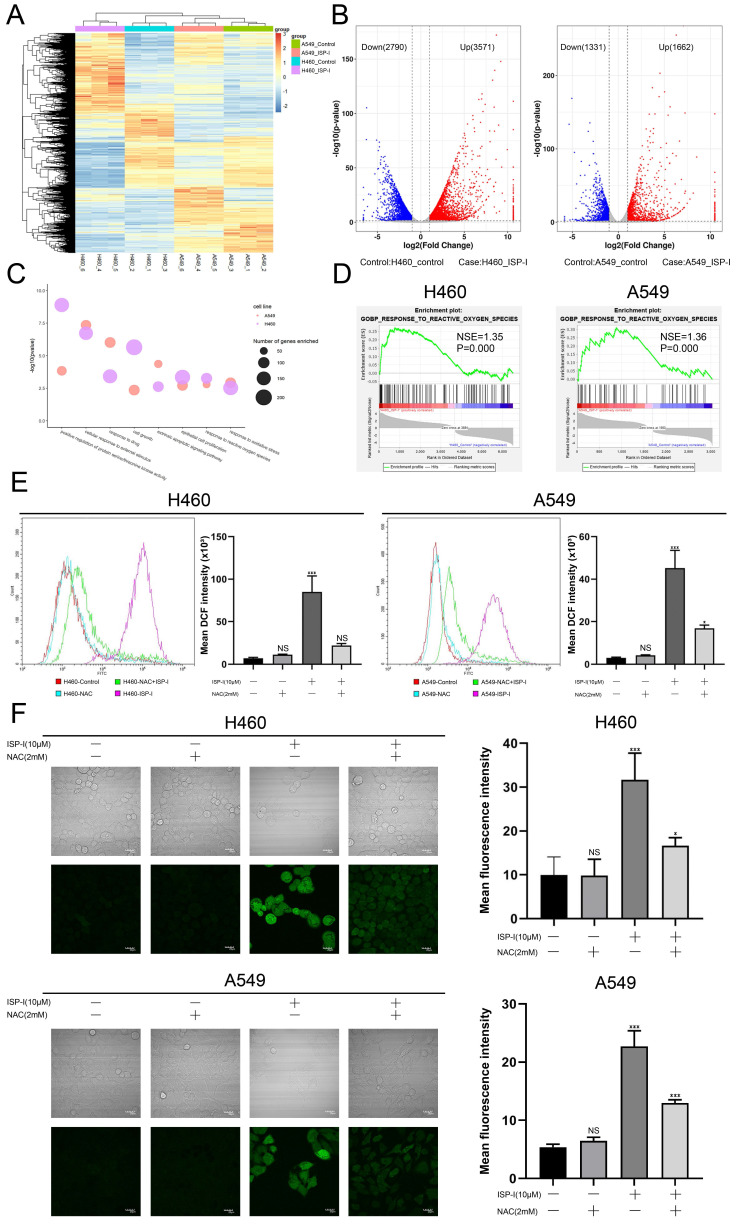
**ISP-I significantly enhanced the intracellular ROS levels in NSCLC cells. (A)** The cluster heat map of DEGs expression patterns of H460 and A549 after treatment with ISP-I vs. control as detected by RNA-Seq (up-regulated, red; down-regulated, blue).** (B)** Volcano plots showed DEG number of H460 and A549 after ISP-I treatment vs. control (up-regulated, red; down-regulated, blue; unchanged, black). **(C)** List of enriched GO_BP terms. **(D)** GSEA enrichment plots after ISP-I treated, NES and P were shown in each plot.** (E)** The relative fluorescence intensity for ROS generation by flow cytometry in H460 and A549 after ISP-I (10μM, 48h) with or without NAC (2 mM) treatment for 48h. **(F)** Confocal microscopy images of intracellular ROS generation in H460 and A549. Treatment protocols were the same as described in Fig. 3E. The mean fluorescence intensity for ROS generation was analyzed by Image J. Scale bars=20μm. ns p > 0.05, * p < 0.05 and *** p < 0.001.

To explore the possible mechanisms of ISP-I-induced apoptosis and G2/M arrest, we performed RNA-seq comparisons of NSCLC cells after ISP-I (10μM) treatment for 48h. The results demonstrated that 3571 genes were up-regulated and 2790 genes were down-regulated after ISP-I treatment in H460, similar results were observed in A549 (Fig. 3A, 3B). We analyzed DEGs by Gene Ontology (GO) enrichment analysis. Here, we focused on the BP. All possibly relevant BP terms were presented in the bubble chart and ROS related terms (e.g., GO: 0006979, GO: 0000302, GO: 0034599) were significantly enriched (Fig. 3C). GSEA was also performed. The results demonstrated that response to ROS was markedly positive regulation in H460 and A549 treated with ISP-I (H460: normalized enrichment score (NES) = 1.35, normalized p-value (P) = 0.000; A549: NES = 1.36, P = 0.000) (Fig. 3D). According to the above analysis, we hypothesized that ROS accumulation was pivotal biological processes triggered by ISP-I. Next, we identified excess intracellular ROS contents through flow cytometry (Fig. 3E) and confocal microscopy (Fig. 3F). The data indicated that treatment with ISP-I (10μM) for 48h significantly increased cellular ROS levels in H460 and A549 cells, and 2 mM ROS scavenger NAC could effectively decrease ROS accumulation.

**Fig 4:**
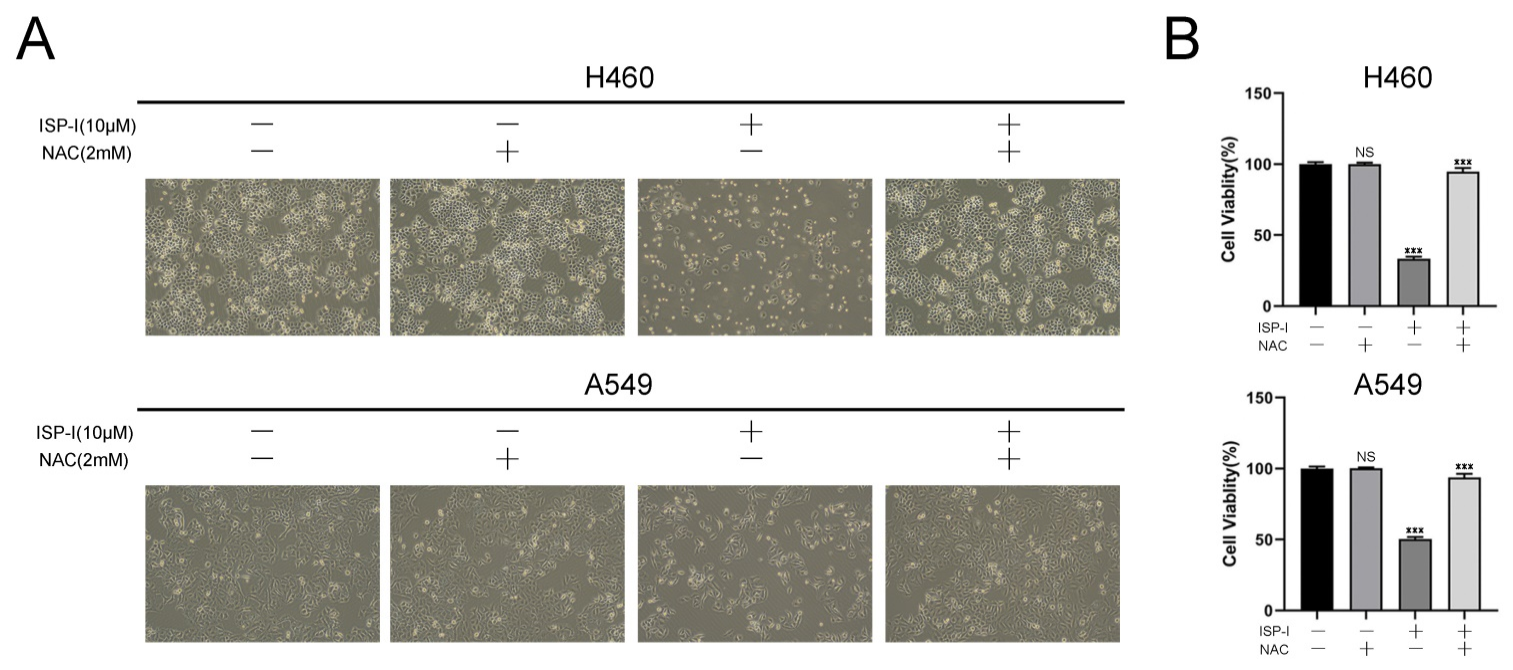
** The anti-tumor effect of ISP-1 depended on ROS accumulation. (A)** Changes in the morphology and cell numbers of H460 and A549 after indicated treatments as imaged by inverted microscope (magnification, 100×). **(B)** Viabilities of H460 and A549 cells after ISP-I (10μM, 48h) treatment with or without NAC (2 mM) were assessed by CCK-8 assay. *** p < 0.001.

ROS accumulation could trigger cell death in many aspects 26. We next assessed whether ROS was involved in ISP-I-induced NSCLC growth inhibition. As shown in Fig. 4A and 4B, the changes of cell morphology (Fig. 4A) and viability (Fig. 4B) after ISP-I treatment in H460 and A549 were remarkably reversed by the addition of NAC. Taken together, ROS accumulation is required for ISP-I-mediated tumor growth inhibition in NSCLC cells.

### The ROS-mediated PI3K/AKT signaling pathway was involved in the anti-tumor performance of ISP-I

**Fig 5:**
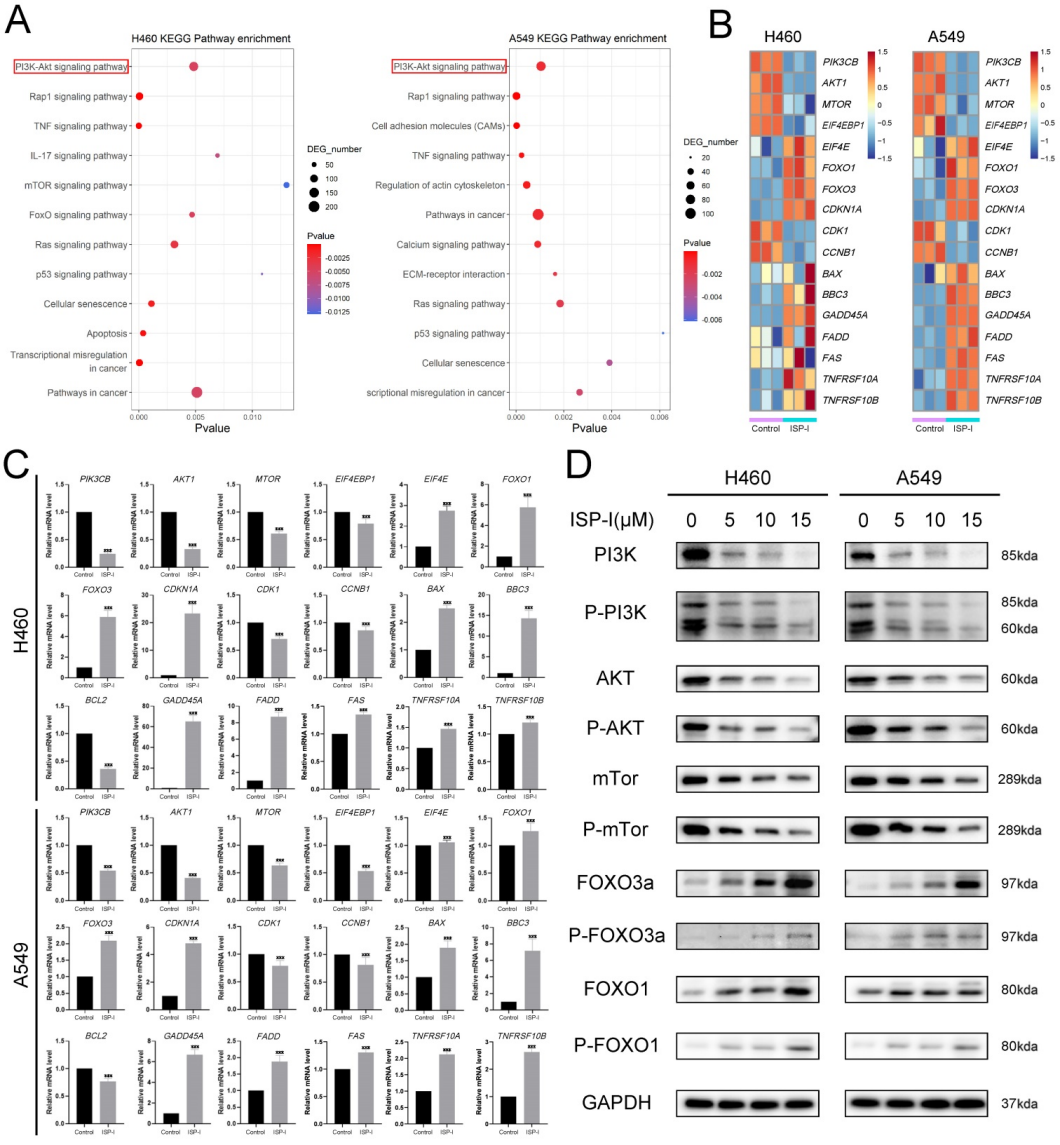
** The PI3K/AKT signaling pathway was significantly suppressed. (A)** The significantly enriched pathways of DEGs related with NSCLC growth were shown in the bubble chart by KEGG pathway analysis. **(B)** A heat map from RNA-seq dataset demonstrated the mRNA levels of crucial genes involved in PI3K/AKT signaling pathway in H460 and A549 after ISP-I treatment for 48h vs. control. (high expression, red; low expression, blue).** (C)** The mRNA expression levels of genes mentioned above was assessed by qRTPCR. **(D)** The protein expression levels of total PI3K, total AKT, total mTOR, total FOXO3a, total FOXO1, p-PI3K, p-AKT, p-mTOR, p-FOXO3a and p-FOXO1 were examined after ISP-I treatment for 48h in H460 and A549 by western blotting. *** p < 0.001.

To find the detailed mechanism underlying ROS-mediated tumor inhibition, we analyzed DEGs by KEGG pathway. Several pathways could be affected by ISP-I and one of the most enriched pathways was the PI3K/AKT signaling pathway (Fig. 5A). This pathway is notably ROS-regulated and performs important functions in cancer cell proliferation and apoptosis 27. Here, we identified that PI3K/AKT signaling pathway was significantly suppressed by ISP-I. A heat map showed the mRNA level alteration of several related genes, including PIK3CB (PI3K), AKT1 (AKT), MTOR, EIF4EBP1 (4eBP1), EIF4E (eIF-4E), FOXO1, FOXO3, CDKN1A (p21), CDK1 (cdc2), CCNB1 (cyclinB1), GADD45A (GADD45), BBC3 (Puma), BAX, Fas (Apo1, CD95), TNFRSF10A (Apo2, DR4), TNFRSF10B (DR5) and FADD, which were the crucial genes involved in PI3K/AKT signaling pathway and directly led to apoptosis and G2/M arrest (Fig. 5B). Next, we validated the expression of key differential genes mentioned above by qRTPCR, and the results were consistent with the RNA-seq analysis (Fig. 5C). We also used western blotting to verify PI3K/AKT signaling pathway inhibition. In H460 and A549, the expression levels of total PI3K, total AKT, total mTOR, p-PI3K, p-AKT and p-mTOR were obviously decreased after 48h treatment with ISP-I in a dosage-dependent manner, whereas total FOXO1/3a and p-FOXO1/3a were up-regulated (Fig. 5D).

**Fig 6:**
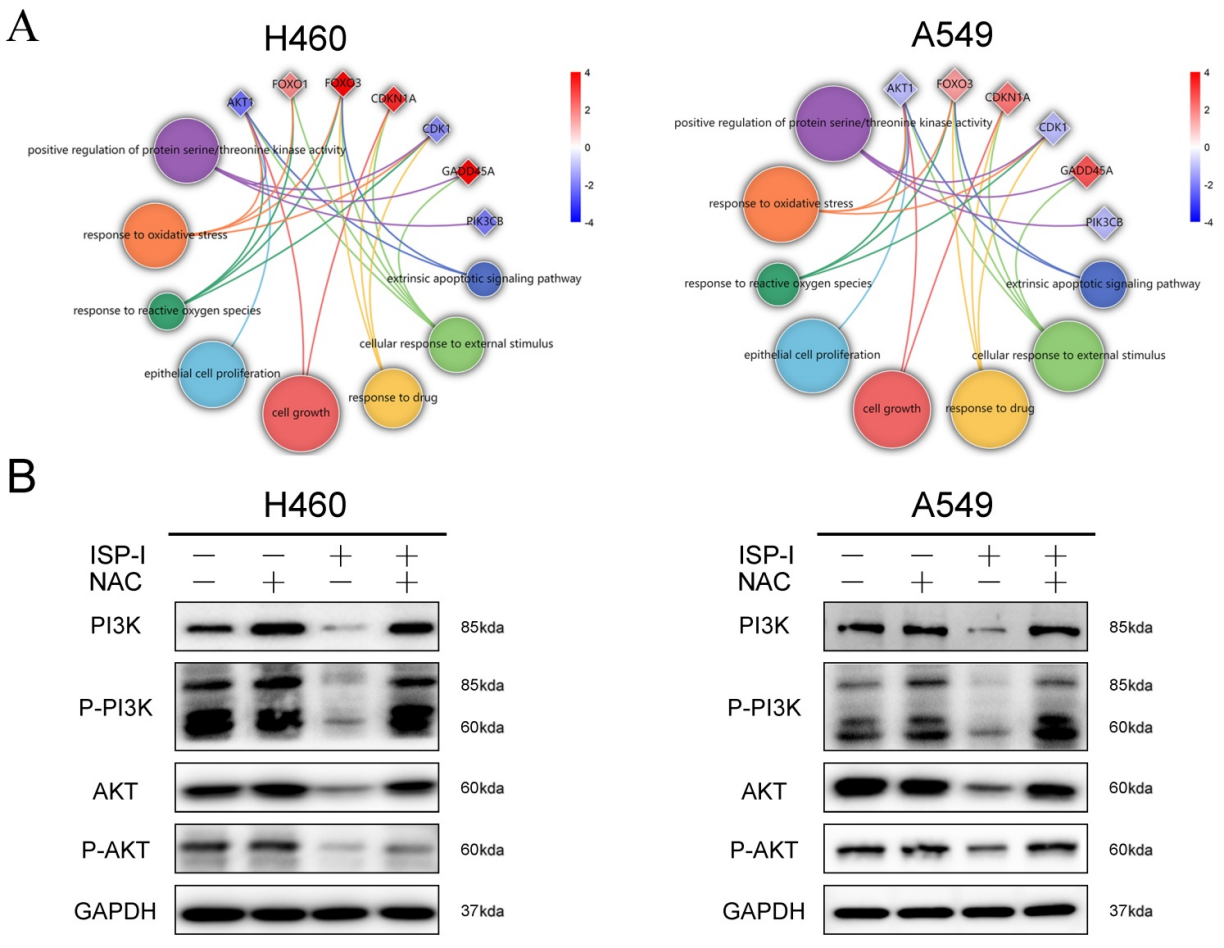
** PI3K/AKT signaling pathway inactivation was ROS-dependent. (A)** Circle plots from RNA-seq data revealed the relationship between enriched BP terms, including ROS related terms, and several essential genes involved in PI3K/AKT pathway. **(B)** The protein expression levels of PI3K, p-PI3K, AKT and p-AKT in H460 and A549 after ISP-I (10μM, 48h) treatment with or without NAC (2 mM) by western blotting.

ROS accumulation has been implicated as an upstream regulator in inhibition of PI3K/AKT pathway 28, 29. Circle plots from RNA-seq data depicted that the enrichment GO_BP terms, especially ROS related, were closely correlated with several essential genes involved in PI3K/AKT pathway (Fig. 6A). We further treated H460 and A549 with 10μM ISP-I alone or in combination with 2mM NAC for 48h. The results showed that compare to single ISP-I treatment group, the expression levels of PI3K, p-PI3K, AKT and p-AKT in H460 and A549 were restored after ISP-I + NAC treatment, which suggested that PI3K/AKT pathway inactivation was mediated by ROS accumulation (Fig. 6B). In conclusion, these above findings validated that the PI3K/AKT signaling pathway was involved in ROS-mediated tumor inhibition.

### ISP-I inhibited growth of tumor in vivo

**Fig 7:**
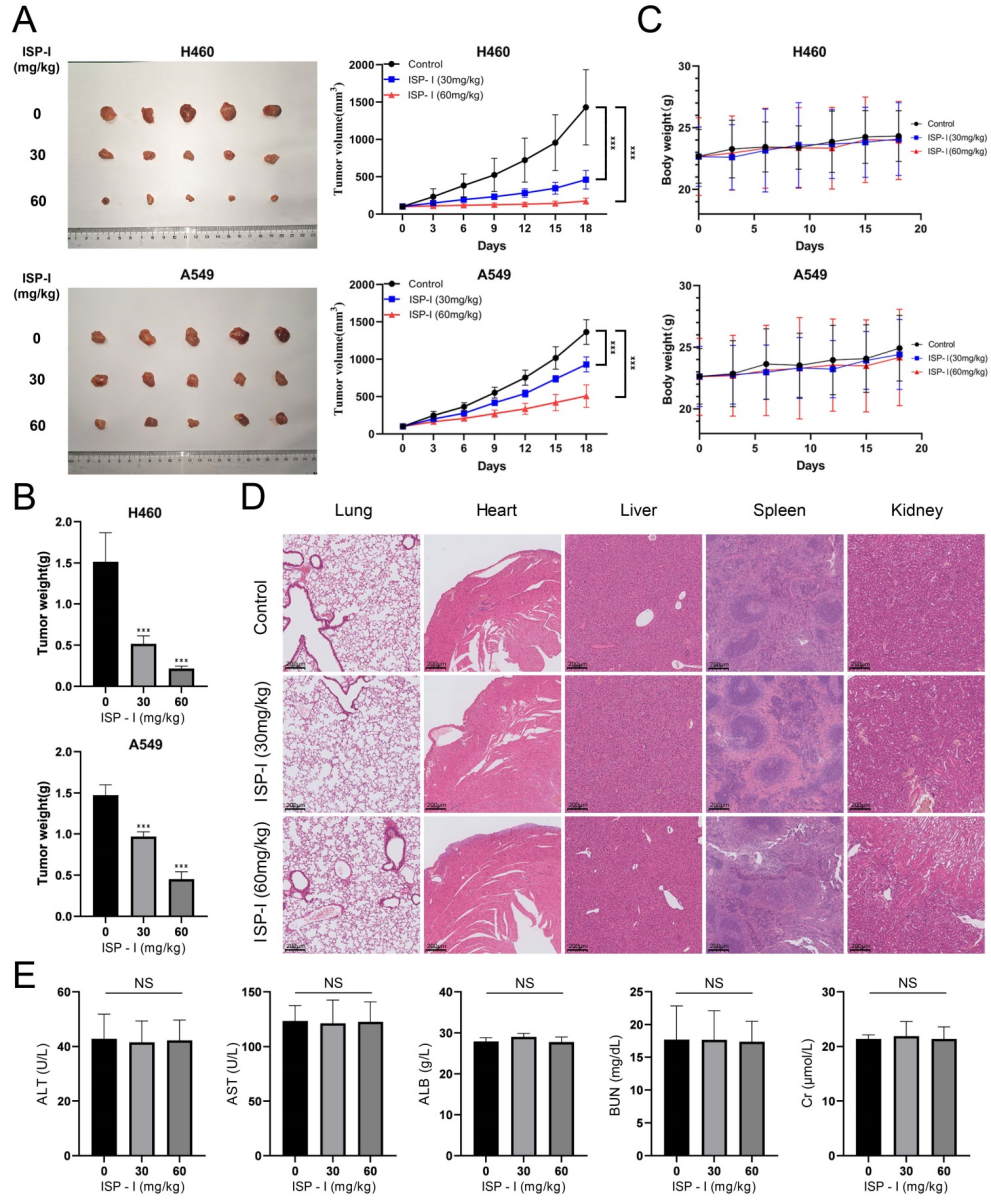
** ISP-I possessed a favorable tumor-suppressive effect as well as a good safety profile in vivo. (A)** Xenograft tumors in vehicle-treated group and ISP-I-treated groups (30mg/kg, 60mg/kg) after 18 days' treatment. Tumor volumes were calculated every 3 days to plot tumor growth curve. **(B)** Tumors weight in vehicle-treated group and ISP-I-treated groups (30mg/kg, 60mg/kg) at the end of experiments. **(C)** Body weight curve in nude mice. **(D)** The HE staining images of major organs in mice after vehicle- and ISP-I-treatment (30mg/kg, 60mg/kg).** (E)** Liver and kidney function indicators between blank control group and ISP-I-treated groups. n=5, *** p < 0.001.

In vivo effect of ISP-I on NSCLC was determined via intragastric administration in a tumor-transplanted nude mouse model established by subcutaneously injecting H460 and A549 cells. After treating with ISP-I for 18 days at doses of 30 and 60 mg/kg, the tumor volume and weight were both significantly inhibited compared to vehicle-treated group (Fig. 7A-7B), while the body weight of mice was stable with a slight upward trend (Fig. 7C). Furthermore, we also generated orthotopic lung tumors to directly detect the efficacy of ISP-I against NSCLC in situ. After 18 days of treatment, the number and size of tumors in the lung of each experimental group (30 and 60 mg/kg) were significantly less than those in control group (Supplementary Fig. S2).

To further investigate whether ISP-I had potential cytotoxic effects on normal tissues, organs and blood samples of various treated groups (0mg/kg, 30mg/kg, 60mg/kg) were collected after oral administration for 18 consecutive days. HE staining of major organs exhibited no significant pathological changes, which revealed ISP-I was no noticeable major organ-related toxicities (Fig. 7D, Supplementary Fig. S3). Blood biochemical tests were carried out as well. The results showed that the liver and kidney function indicators between blank control group and ISP-I-treated groups were no statistical differences and remained at normal levels (Fig. 7E). In addition, to further evaluate ISP-I's effect on the more proliferating organs of the body, we added IHC staining analysis of proliferation markers Ki67 and apoptosis markers cleaved-caspase-3 of stomach, small intestine, skin, and eye. The results indicated that treatment with ISP-I did not exert an effect on the more proliferating organs in nude mice (Supplementary Fig. S3). To sum up, no significant tissue toxicity and inflammation were detected after treatment with ISP-I at the maximum daily dose of 60mg/kg. The above results indicated that ISP-I possesses a favorable tumor-suppressive effect as well as a good safety profile.

**Fig 8:**
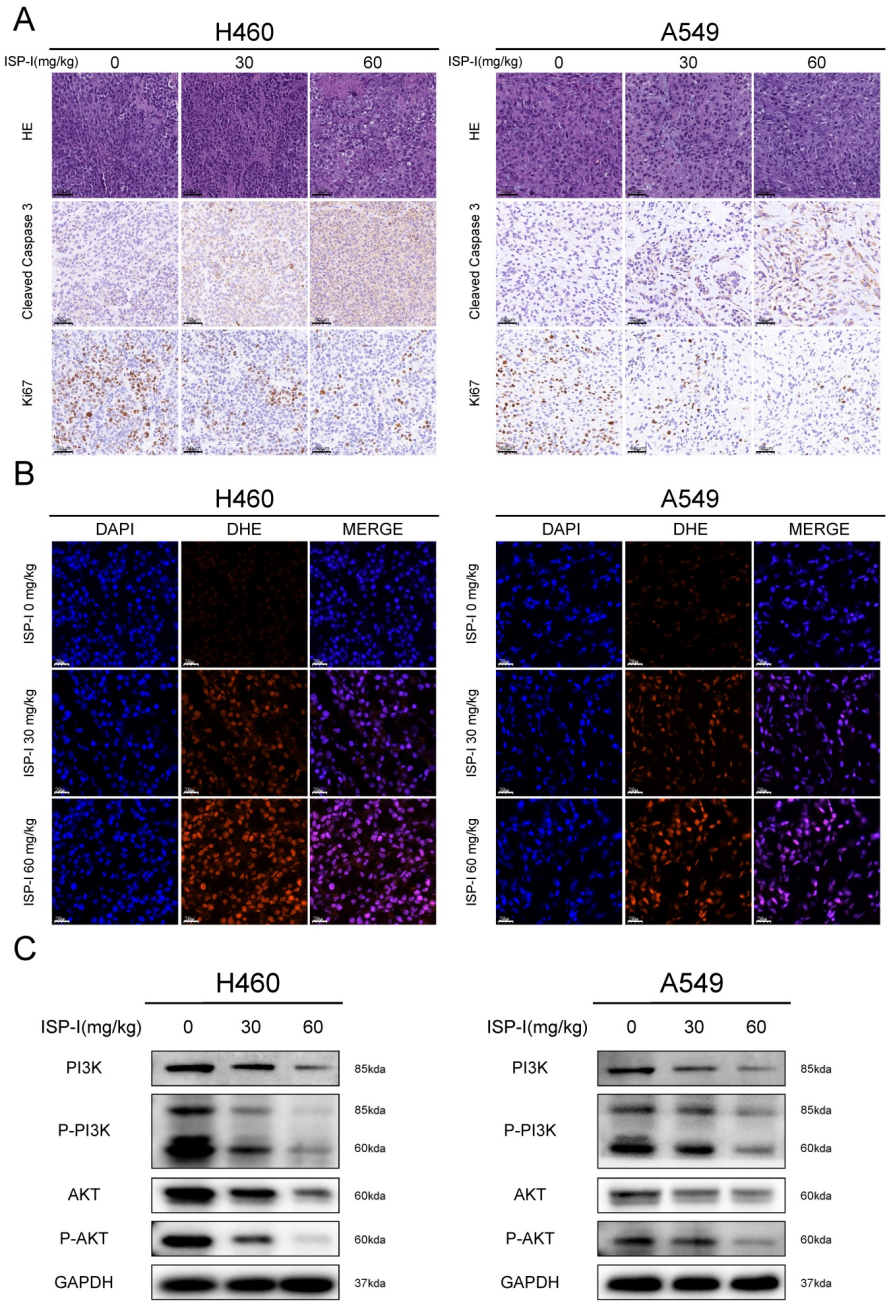
** ISP-I inhibited tumor proliferation and promoted tumor apoptosis in vivo by generation of ROS resulting in PI3K/AKT pathway inhibition. (A)** HE staining of tumor tissues and immunohistochemistry of Ki67, cleaved Caspase3 in vehicle-treated group and ISP-I-treated groups (30mg/kg, 60mg/kg). **(B)** Fluorescent microscopic observation of tumor tissues stained with DHE, which reflected ROS levels. **(C)** The protein expression of PI3K, p-PI3K, AKT and p-AKT in vehicle-treated group and ISP-I-treated groups (30mg/kg, 60mg/kg) by western blotting.

We next verified the mechanism of ISP-I's tumor-suppressing function in vivo. IHC was used to investigate the proliferation and apoptosis of xenograft tumor. ISP-I treated tumor tissues showed high cleaved Caspase-3 expression and low proliferation marker Ki-67 expression (Fig. 8A). DHE staining could be applied to detect the in situ ROS levels in frozen tissue sections 30. In our research, we found that DHE was markedly increased in ISP-I treated tumors compared with the controls (Fig. 8B), indicating excessive ROS accumulation. In western blotting of xenograft samples, ISP-I treatment led to a decrease in p-PI3K, PI3K, AKT, p-AKT levels compared with vehicle-treated group (Fig.8C). In conclusion, ISP-I significantly inhibited tumor growth in vivo by generation of ROS resulting in inhibition of PI3K/AKT pathway. These results were consistent with that in vitro.

## Discussion

The issues of toxic side effect and multidrug resistance (MDR) remain a challenge in the treatment and clinical management of NSCLC recently 31. It is crucial to find some small molecules with good therapeutic potentials and low toxicity to act as a novel drug candidate for comprehensive treatment of NSCLC 24. Repurposing old drugs in treating other diseases is a technique with many advantages including time and cost savings, since the safety and toxicity of candidate drugs have already been clearly studied 17. Carrimycin is a Chinese Food and Drug Administration (CFDA)-approved macrolide antibiotic 32. Here, we firstly isolated the active component of carrimycin, ISP-I, and verified its satisfying anti-NSCLC effect both in vitro and in vivo. Moreover, we identified that ISP-I possessed an excellent safety profile, based on its less toxic to human bronchial epithelial cell BEAS-2B in vitro and no significant weight loss or organ toxicities to oral administration mice in vivo. Therefore, we proposed that ISP-I represented a new therapeutic agent for NSCLC.

Apoptosis and cell cycle arrest are two main reasons in suppression of tumor growth 33, 34. In our study, we found that ISP-I could cause apoptosis and G2/M arrest. Apoptosis can be triggered via two canonical pathways: the intrinsic mitochondrial pathway, and the extrinsic apoptotic pathway 35. PUMA, BAX and BCL-2 are prominent members of BCL-2 family, which can trigger mitochondria-induced endogenous apoptosis through BCL-2/Caspase-9/Caspase-3 cascade reaction 23. Sequential activation of caspase-8 is the central event in the execution-phase of extrinsic apoptosis, and followed by the direct activation of effector caspases3 36, 37. Our results indicated that ISP-I led to both intrinsic and extrinsic apoptosis through caspase-dependent cell signaling cascades.

Cell cycle deregulation is a common mechanism of tumorigenesis and can help tumor cells escape from tumor-suppressive pathways 38, 39. Three major cell cycle checkpoints (G1/S, S phase and G2/M checkpoints) are pivotal steps in the process of cell cycle 40, which can be validated as targets for anti-tumor drugs 41. Our results showed that ISP-I suppressed cell proliferation associated with G2/M arrest. Activation of the cyclin B1/cdc2 complex is central to facilitate G2/M cell cycle transition, mainly mediated by dephosphorylation of cdc2 42, 43. P21, as a vital member of CDK inhibitor, suppresses cyclin B1/cdc2 complex activity by interacting with many important proteins, including cdc2 and GADD45A 44, thereby inhibiting the G2/M transition in various cancer types both p53-dependent and p53-independent 45, 46. In our study, we demonstrated that ISP-I markedly up-regulated the expression of p-cdc2 and p21, followed by decreasing the expression of cyclin B1 and cdc2. Overall, our results revealed that ISP-I could act as a cell cycle blocker regardless whether p53 whether present or not.

As is well known, induction of apoptosis and G2/M arrest is tightly controlled by various biological processes and regulatory molecules 47. Among them, excessively accumulated ROS is involved in tumor growth inhibition and acts as an important mediator which can cause apoptosis and cell cycle arrest via multiple routes 48, 49. According to our results, we found that ISP-I could significantly enhance ROS accumulation, whilst the ROS scavenger NAC almost entirely reversed the antitumor activity of ISP-I in NSCLC cells. Hence, ISP-I suppressed NSCLC cell growth through ROS over-accumulation, which may serve as a critical upstream regulator to the anti-cancer potentials of ISP-I. The baseline ROS levels of tumor cells are generally much higher than normal cells and increase along with the tumor progression due to its vigorous cell metabolism and proliferation 50, 51. These findings indicate that cancer cells may be more sensitive to agents that induce further ROS accumulation compared to normal cells 52, which provide a specific therapeutic window for tumor targeting. Taken together, ISP-I may act as a novel ROS inducer for anti-NSCLC treatment.

The PI3K/AKT pathway inhibition has been identified to strongly associate with ROS accumulation and involved in ROS-induced tumor suppression 53, 54. In our research, we confirmed that the PI3K/AKT pathway was significantly repressed and could be rescued efficiently by NAC. Our results therefore revealed the detailed mechanism underlying ISP-I-mediated cell growth inhibition caused by ROS over-accumulation. Aberrant activation of the PI3K/AKT pathway is prevalent across various cancer types, and often associates with the tumor progression and poor prognosis 55, 56. Activated AKT, as a key downstream effector of the P13K signaling pathway, can modulate numerous substrates' function involved in a variety of biological activities including cell growth, proliferation, death and metabolism (bad, procaspase-9, p21Cip/Waf1, mTOR, FOXOs etc.) 57. FOXOs are discovered as a tumor suppressor through activating G2/M arrest related genes CDKN1A and GADD45A, apoptosis related genes FAS, DR4 and DR5, respectively 58-62. FAS, DR4 and DR5 are important cell surface death receptors, which recruit FADD to induce death-inducing signaling complex (DISC) formation and subsequent caspase-8 activation 63. Activation of caspase 8 is an essential marker for extrinsic apoptosis. mTOR, which is another important downstream protein of PI3K/AKT signaling pathway, modulates cell growth and metabolism 64. According to the RNA-seq data and qRTPCR, we confirmed that PI3K/AKT pathway is inactivated through ROS accumulation caused by ISP-I, which in turn induced corresponding changes in numerous downstream genes related to apoptosis and G2/M arrest. The diversity of alterations in PI3K/AKT pathway (PIK3CB, AKT1, MTOR, FOXOs, CCKN1A etc.) provides several molecular therapeutic targets 65 and ISP-I exhibits an excellent potential of multiple-target inhibitions. These results indicated that ISP-I was a promising anti-tumor agent due to this unique multi-target ability, which may compensate for the deficiency of traditional single-target agents 66 and decrease the probability of chemotherapy resistance.

KEAP1 loss-of-function mutations occur in various cancer types and can decrease NRF2 degradation to activate the KEAP1/NRF2 antioxidant response pathway, which confers a poor prognosis 67. 17% of lung adenocarcinomas possess KEAP1/NRF2 alterations against oxidative stress 68. Some studies have demonstrated that combined aberrant activation of PI3K and KEAP1/NRF2 pathways can substantially promote NSCLC development and is often linked with chemotherapy drug resistance 69. In our study, we used NSCLC cell lines A549 and H460, which carried KEAP1 inactive homozygous mutations 70. The excellent anti-tumor effect shows that ISP-I provide a new therapeutic option targeting to NSCLC patients carrying KEAP1 mutation through excessive ROS accumulation and play dual roles as a PI3K/AKT pathway inhibitor simultaneously. In addition, numerous studies have shown that compounds leading to even higher oxidative stress can sensitize MDR cancer cells to certain chemotherapeutic drugs or even induce MDR cancer cells death, regardless of the underlying mechanisms of MDR 71. As a result, ISP-I is considered a promising alternative to overcome the drug resistance of MDR cancer cells. Moreover, ROS inducing agents are reported to be used in combination with traditional therapies, immunotherapy or other pro-oxidant agents to exert synergistic effects 9, 10, 54, 72. Therein, combined ISP-I with traditional chemoradiotherapy, immunotherapy or other pro-oxidant agents such as doxorubicin, β-phenylethyl isothiocyanates can probably exhibit a higher tumor killing activity and further increase their clinical efficacy along with a reduction of side effects to improve survival. These need to be further studied.

To sum up, our study shows that ISP-I, the active component of CFDA-approved macrolide antibiotic carrimycin, induces apoptosis and G2/M arrest through ROS-mediated PI3K/AKT signaling pathway inhibition, ultimately leading to NSCLC cell death. ISP-I is a novel ROS inducer and may be a promising candidate with low biological toxicity and multi-target ability for anti- NSCLC treatment.

## Supplementary Material

Supplementary figures and tables.

## Abbreviations

ISP-I: isovalerylspiramycin I
NSCLC: non-small cell lung cancer
RTKs: multiple receptor tyrosine kinases
PEG: polyethylene glycol
DHE: dihydroethidium
NAC: N-acetyl-l-cysteine
FBS: fetal bovine serum
DEGs: differentially expressed genes
GO: Gene Ontology
BP: biological processes
KEGG: Kyoto Encyclopedia of Genes and Genomes
MFI: mean fluorescence intensity
GSEA: Gene Set Enrichment Analysis
qRTPCR: quantitative real-time PCR analysis
HE: hematoxylin and eosin
IHC: immunohistochemistry
ALT: alanine transaminase
AST: aspartate transaminase
ALB: albumin
BUN: blood urea nitrogen
Cr: creatinine
MDR: multidrug resistance
CFDA: Chinese Food and Drug Administration
