# Supplementary material for: Isovalerylspiramycin I suppresses non-small cell lung carcinoma growth through ROS-mediated inhibition of PI3K/AKT signaling pathway
Source: Int J Biol Sci. 2022 May 21;18(9):3714–30. doi: 10.7150/ijbs.69989 (PMC9254468; doi:10.7150/ijbs.69989)
Supplement: Supplementary file 1 — Supplementary figures and tables. [file ijbsv18p3714s1.pdf]

Supplementary Materials for

IsovalerylspiramycinⅠsuppresses non-small cell lung carcinoma growth through ROS-mediated inhibition of PI3K/AKT signaling pathway

Zeyu Liu, Moli Huang, Yue Hong, Shaoyang Wang, Yongle Xu, Cheng Zhong, Jingyuan Zhang, Zhengping Zhuang*, Shan Shan*, Tao Ren*

*Corresponding author. Email: liuyuanrentao@sjtu.edu.cn (Tao Ren), shanshan_shcn@126.com (Shan Shan), zpzhua@hotmail.com (Zhengping Zhuang)

The PDF file includes:

Fig. S1. ISP-I possessed a vigorous anti-NSCLC effect in vivo.

Fig. S2. ISP-I exhibited a favorable tumor inhibition effect in orthotopic lung tumor model.

Fig. S3. Safety assessment of the more proliferating organs after ISP-I treatment.

Table S1. Antibodies used in this study.

Table S2. Primers' sequences used for qRTPCR.

Fig. S1

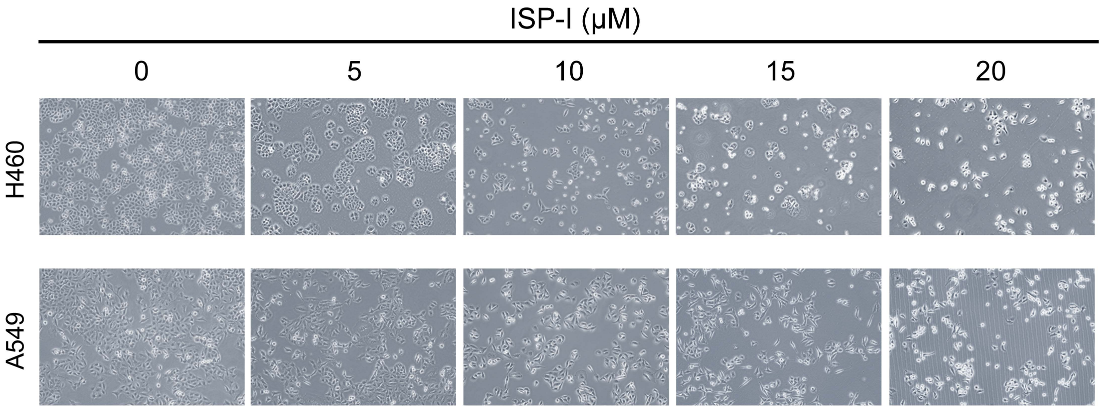

Fig. S1. ISP-I possessed a vigorous anti-NSCLC effect in vivo. Human NSCLC cell lines (H460 and A549) were treated with different doses of ISP-I or DMSO (control) for 48 h. A Nikon Eclipse Ts2 inverted microscope (100× magnification) was used to observe and image cell morphology and numbers.

Fig. S2

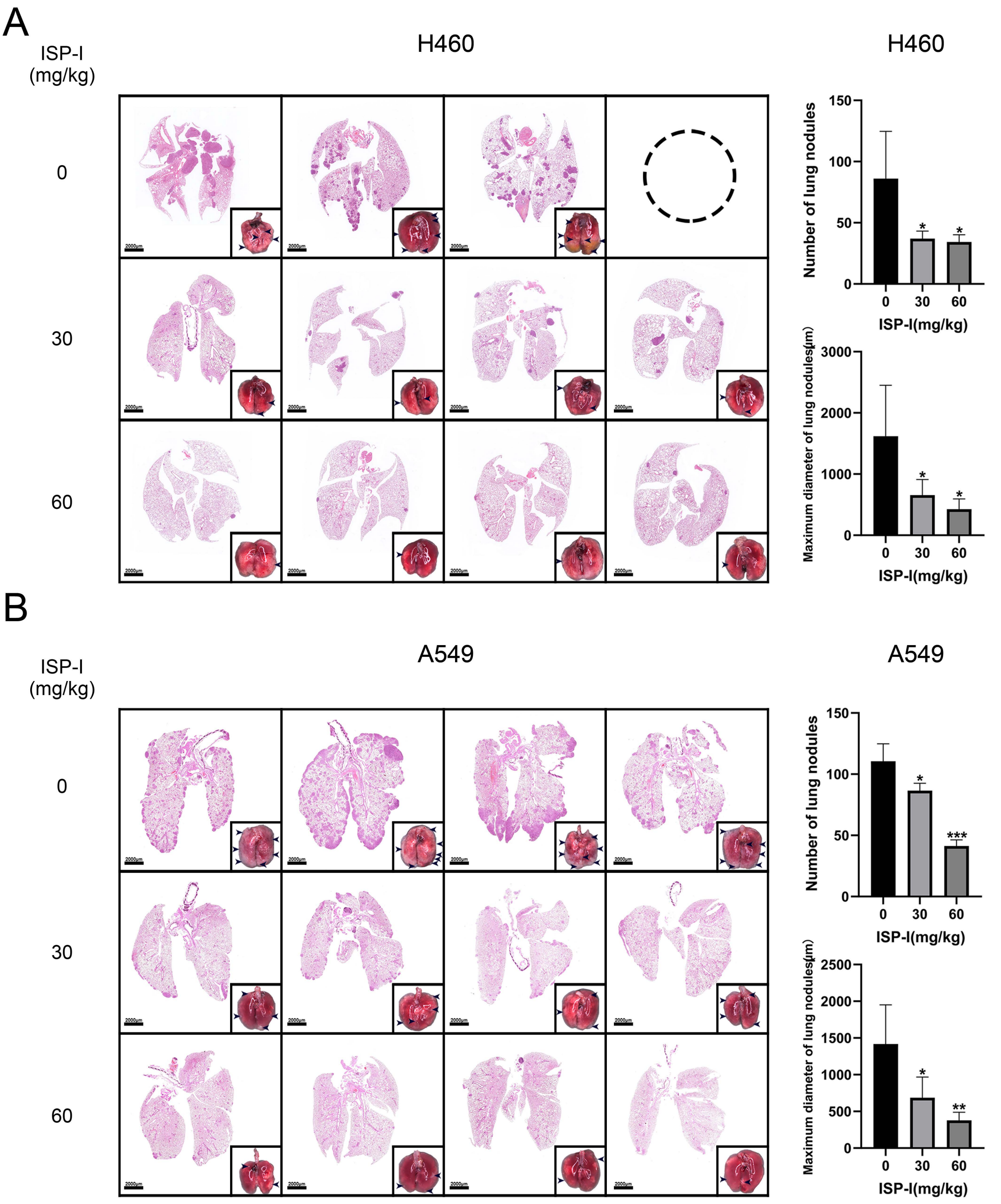

Fig. S2. ISP-I exhibited a favorable tumor inhibition effect in orthotopic lung tumor model. ISP-I (30 or 60 mg/kg, experimental groups) or vehicle (water containing PEG, vegetable oil and Tween-80, control group) were administered daily by oral gavage in nude mice with orthotopic lung tumors formed by H460 **(A)** and A549 **(B)**. After 18 days, the mouse lungs were

removed for gross observation and HE staning (Left panels). Number and maximum diameter of lung nodules were measured further (Right panels). Data are expressed as mean ± standard deviation，n=4 unless one mouse in the H460-control group died during the experiment (black circle).

Fig. S3

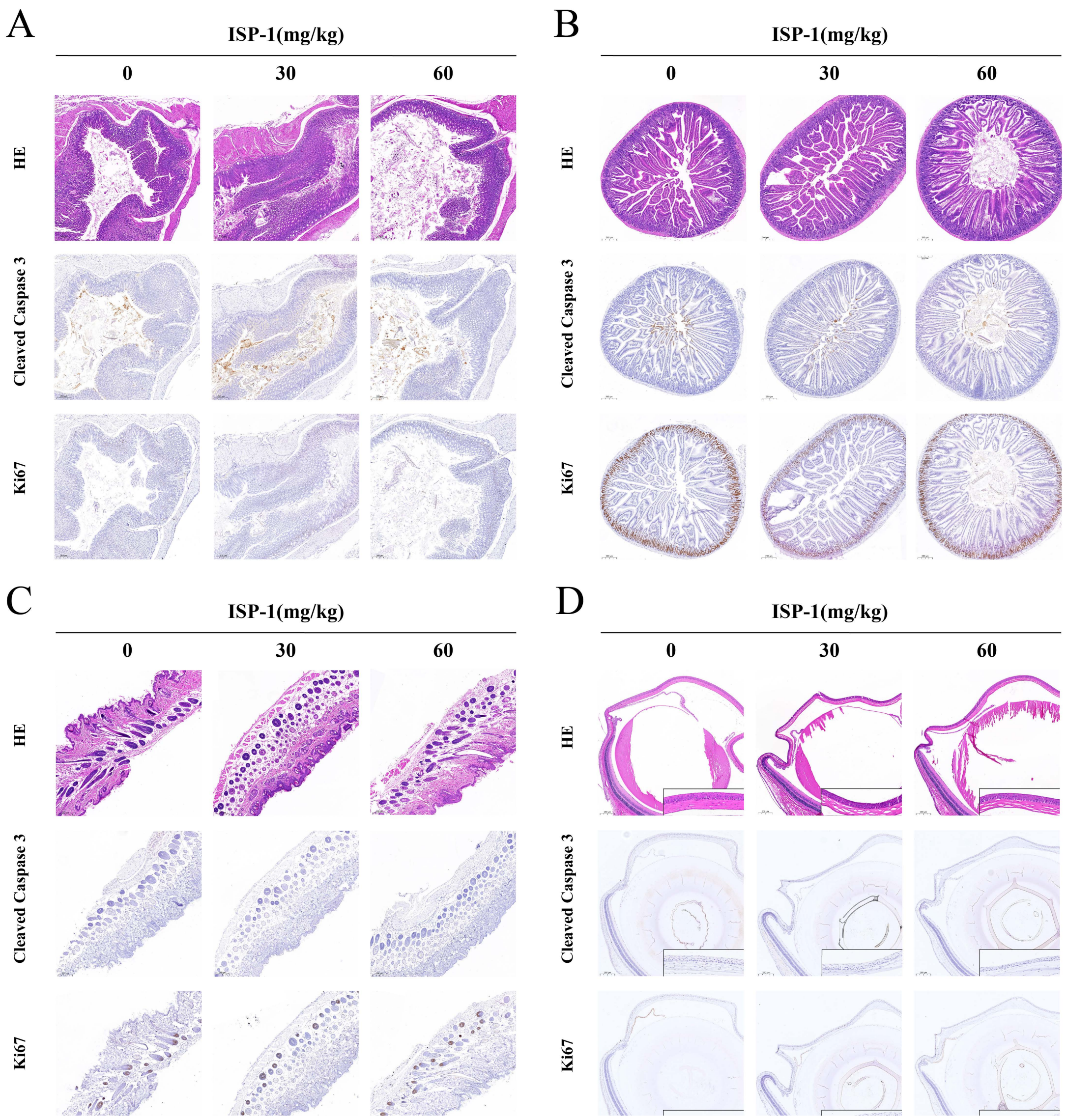

Fig. S3. Safety assessment of the more proliferating organs after ISP-I treatment. After 18 days treatment of vehicle or ISP-I (30 or 60 mg/kg), the more proliferating organs, including stomach (A), small intestine (B), skin (C) and eyes (D), were removed for HE and IHC staining of Ki67 and cleaved Caspase3. No pathological changes on tissue morphology, proliferative activity and apoptosis were found in ISP-I treated groups, compared with control group.

Table S1. Antibodies used in this study.

Antibodies	Supplier	Cat no.
cleaved caspase-3(Asp175)	Cell Signaling Technology	#9664
cleaved caspase-8(Asp374)	Cell Signaling Technology	#9496
cleaved caspase-9(Asp330)	Cell Signaling Technology	#52873
p21 Waf1/Cip1	Cell Signaling Technology	#2947
cyclin B1	Cell Signaling Technology	#4138
cdc2	Cell Signaling Technology	#9116
Phospho-cdc2 (Tyr15)	Cell Signaling Technology	#9111
PI3 Kinase p85	Cell Signaling Technology	#4257
Phospho-PI3 Kinase p85(Tyr458)/p55(Tyr199)	Cell Signaling Technology	#4228
Akt	Cell Signaling Technology	#4691
phospho-AKT(Ser473)	Cell Signaling Technology	#4060
mTOR	Cell Signaling Technology	#2983
phospho-mTOR (Ser2448)	Cell Signaling Technology	#5536
FoxO3a	Cell Signaling Technology	#2497
phospho-FoxO3a (Ser253)	Cell Signaling Technology	#9466
FoxO1	Cell Signaling Technology	#2880
phospho-FoxO1 (Ser256)	Cell Signaling Technology	#9461
Bcl-2	Abcam	#ab32124
Bax	Abcam	#ab32503

PUMA	Abcam	#ab9643
GAPDH	Abcam	#ab8245
Ki67	Abcam	#ab16667
HRP-conjugated Goat Anti-Rabbit IgG (H+L)	ABclonal	# AS014
HRP-conjugated Goat Anti-Mouse IgG (H+L)	Abclonal	# AS003

Table S2. Primers' sequences used for qRTPCR.

Gene	Species	Primer sequence
PIK3CB	Human	Forward: GACTTTGCGACAAGACTGCC
		Reverse: AATCTGAAGCAGCGCCTGAA
AKT1	Human	Forward: CAGGATGTGGACCAACGTGA
		Reverse: AAGGTGCGTTCGATGACAGT
MTOR	Human	Forward: TGCCAATGAGAGGAAAGGTG
		Reverse: CCAATTCGGGTGGCATTC
EIF4EBP1	Human	Forward: TGTGACCAAAACACCCCCAA
		Reverse: TGGTAGTGCTCCACACGATG
EIF4	Human	Forward: CAAACCTGCGGCTGATCTC
		Reverse: TCCCACATAGGCTCAATACCA
FOXO1	Human	Forward: GTGGAGATCGACCCGGACTT
		Reverse: CCGAGTTGGACTGGCTAAAC
FOXO3	Human	Forward: AGAAGTTCCCCAGCGACTTG
		Reverse: TCCCCACGTTCAAACCAACA
CDKN1A	Human	Forward: CTGCCCAAGCTCTACCTTCC
		Reverse: CGAGGCACAAGGGTACAAGA
CDK1	Human	Forward: TGGGGTCAGCTCGTTACTCA
		Reverse: ATGCTAGGCTTCCTGGTTTC
CCNB1	Human	Forward: GGTCGGGAAGTCACTGGAAA
		Reverse: AGCATCTTCTTGGGCACACA

BAX	Human	Forward: CGGGTTGTCGCCCTTTTCTA
		Reverse: GTCCAATGTCCAGCCCATGA
BBC3	Human	Forward: GACCTCAACGCACAGTACGA
		Reverse: ATGGTGCAGAGAAAGTCCCC
BCL2	Human	Forward: TTCCGCGTGATTGAAGACACC
		Reverse: ATCTCCCGGTTATCGTACCCT
GADD45A	Human	Forward: AGAGCAGAAGACCGAAAGGATG
		Reverse: AGGCACAACACCACGTTATC
FADD	Human	Forward: CCTAGACCTCTTCTCCATGCTG
		Reverse: TCTGAGACTTTGAGCTGACGA
FAS	Human	Forward: ACTGTGACCCTTGCACCAAA
		Reverse: AGACAAAGCCACCCCAAGTT
TNFRSF10A	Human	Forward: CTGTTGTTGCATCGGCTCAG
		Reverse: CGAAAGTGGACAGCGAGTCT
TNFRSF10B	Human	Forward: TGACCTCCTTTTCTGCTTGCG
		Reverse: TTCCCCACTGTGCTTTGTACCT
